# Melatonin may increase disease resistance and flavonoid biosynthesis through effects on DNA methylation and gene expression in grape berries

**DOI:** 10.1186/s12870-020-02445-w

**Published:** 2020-05-24

**Authors:** Shiwei Gao, Wanyun Ma, Xinning Lyu, Xiaolei Cao, Yuxin Yao

**Affiliations:** State Key Laboratory of Crop Biology, Collaborative Innovation Center of Fruit & Vegetable Quality and Efficient Production, College of Horticulture Science and Engineering, Shandong Agricultural University, Tai-An, 271018 Shandong China

**Keywords:** Melatonin, Methylome, Transcriptome, Flavonoid biosynthesis, Disease resistance, Grape berries

## Abstract

**Background:**

Melatonin can regulate plant growth, development and biotic responses by causing global changes in gene expression; however, the melatonin-induced changes in gene expression via the modification of DNA methylation remain unclear in plants.

**Results:**

A total of 1,169,852 and 1,008,894 methylated cytosines (mCs) were identified in the control and melatonin-treated grape berries, respectively, and mCs occurred primarily at CG sites, followed by CHG sites and CHH sites. Compared to the control, melatonin treatment broadly decreased methylation levels at CHG and particularly CHH sites in various gene regions. Melatonin treatment generated a total of 25,125 differentially methylated regions (DMRs), which included 6517 DMR-associated genes. RNA-Seq demonstrated that 2479 genes were upregulated, and 1072 genes were repressed by melatonin treatment. The evaluation of the interconnection of the DNA methylome and transcriptome identified 144 genes showing a negative correlation between promoter methylation and gene expression, which were primarily related to biotic stress responses and flavonoid biosynthesis. Additionally, the application of 5́-azacytidine and melatonin led to similar effects on mycelial growth of *B. cinerea*, berry decay rate and flavonoid biosynthesis. Moreover, *EDS1* was used to show that melatonin increased gene expression by decreasing promoter methylation levels.

**Conclusion:**

Our results demonstrated that melatonin broadly decreased DNA methylation and altered gene expression in grape berries. We propose that melatonin increases disease resistance and flavonoid biosynthesis by decreasing the methylation levels of the promoters of the genes involved.

## Background

Grapevine is one of the most important economic fruit crops worldwide. Strategies for improving quality and disease resistance are a key focus of grapevine cultivation. The whole grape berry, including the skin, pulp and seeds, contains melatonin (N-acetyl-5-methoxytryptamine) [1]. Melatonin is a signaling molecule that plays protective roles against biotic and abiotic stresses. Melatonin participates in the regulation of tolerance to various plant diseases. For example, melatonin induces disease resistance to *Botrytis cinerea* in tomato fruit by regulating JA signaling and H_2_O_2_ levels and improves the disease resistance of *Arabidopsis* against pathogen infection via NO signaling [2, 3]. The increase in melatonin content activated by MeRAV1 and MERAV2 enhances plant disease resistance against cassava bacterial blight [4]. Additionally, both exogenous treatment with and endogenous induction of melatonin increase abiotic stress tolerance in many plants. It has been reported that melatonin alleviates salt damage in grapes [5] and enhances the drought tolerance of apple plants [6]. Endogenously increasing melatonin via the overexpression of *ASMT1* significantly enhances the drought tolerance of *Arabidopsis* plants [7].

Melatonin is a multifunctional signaling molecule [8] that promotes grape berry ripening and quality formation via its interplay with other signaling molecules [9]. Melatonin has also been reported to promote tomato and banana ripening and thereby affect fruit quality [10, 11]. Additionally, some other studies have emphasized the role of melatonin in regulating metabolite accumulation in fruits. For example, melatonin treatment enhances the contents of total anthocyanins, phenols and flavonoids in grape berries and wine [12, 13]. Post-harvest treatment with melatonin increases the contents of total phenols and anthocyanins in tomato fruits [10].

The effects of melatonin on stress tolerance and fruit development are largely ascribed to the regulation of gene expression. Global gene expression changes caused by melatonin revealed that melatonin induces salt tolerance via ROS scavenging in rice shoots [14]. Transcriptomic analysis revealed global changes in gene expression related to polyphenol metabolism, carbohydrate metabolism and ethylene biosynthesis and signaling upon melatonin treatment in grape berries [12]. Proteomics data show that 241 proteins are significantly influenced by melatonin in tomato fruits [15]. Arnao and Hernández-Ruiz summarized the genes that are up- and downregulated by melatonin under given physiological conditions, including various abiotic stressors and biotic stressors in different plants [16].

DNA methylation is an important epigenetic modification that plays a very important role in the regulation of multiple biological processes, including stress responses, growth, development and fruit ripening, by modulating gene expression pretranscriptionally [17, 18]. Methylome analysis showed that most DNA methylation-modified genes are transcriptionally altered under Cd stress in rice [19], and a global increase in DNA methylation occurs during orange fruit ripening [20]. Additionally, some specific genes, such as fruit ripening-related RIN and PSY1, show demethylation at their promoters to activate their transcription during fruit ripening [21]. Notably, an increasing number of studies indicate that melatonin alters global DNA methylation levels [22] or the DNA methylation of specific genes [23] in humans. However, the regulation of gene expression by melatonin via the modification of methylation remains unclear in plants.

In this study, the DNA methylome and RNA transcriptome were analyzed in control and melatonin-treated grape berries, and the analysis of their interconnection revealed the key role of melatonin in increasing flavonoid biosynthesis and disease resistance. These functions of melatonin were further verified by determining the melatonin-induced changes in flavonoid contents, the berry decay rate and the levels of expression and promoter methylation of selected genes. These findings provide a new perspective for understanding the role of melatonin in regulating gene transcription in grape berries.

## Results

### Exogenous melatonin treatment increases melatonin content in ‘merlot’ grape berries

**Fig. 1:**
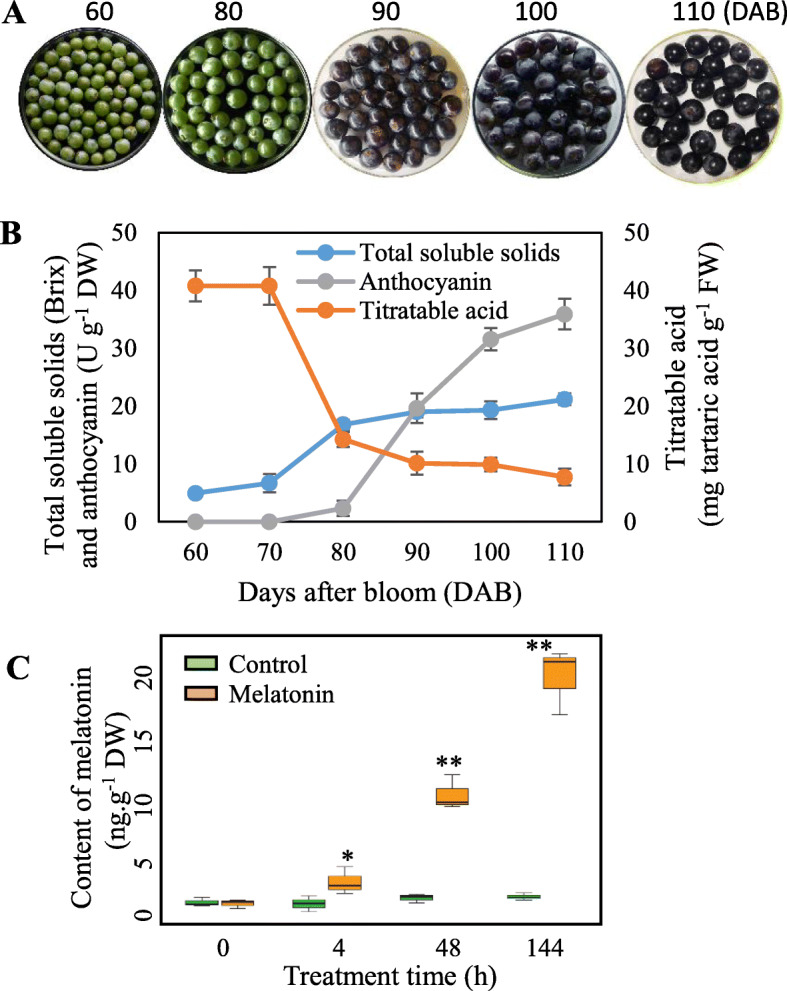
Determination of berry veraison (**a**, **b**) and changes in melatonin content (**c**) in ‘Merlot’ berries under 50 μM melatonin treatment. Berry veraison was indicated by berry coloring, increases in total soluble solids and anthocyanins and decreases in titratable acid. Values represent the means ± SD of three replicates. * Significant difference, *P* < 0.05; ** highly significant difference, *P* < 0.01

Exogenous melatonin treatment was used to increase the melatonin content of grape berries. Veraison (onset of berry ripening) is the key period for ripening regulation using signaling molecules, and melatonin levels begin to increase at veraison [9]. The occurrence of veraison was indicated by the accumulation of sugars and anthocyanins and a decline in titratable acidity. In the ‘Merlot’ grape berries, anthocyanins began to accumulate at 80 days after bloom (DAB), and coloring began at 90 DAB (Fig. 1a, b). Total soluble solids (TSS) and titratable acid continued to accumulate and decrease, respectively, from 80 DAB onward (Fig. 1a, b). Therefore, veraison occurred at approximately 80 DAB, and melatonin treatment was performed at this time point. Treatment with 50 μM melatonin significantly increased the melatonin content of the berries, and increments of 1.24-, 4.45- and 9.02-fold were generated at 4, 48 and 144 h after treatment (HAT), respectively, compared with the control berries (Fig. 1c).

### DNA methylation profiling in control and melatonin-treated ‘merlot’ grape berries

Control and melatonin-treated berries collected at 48 HAT were used for single-base DNA methylation BS-Seq. A total of 877,654,62 and 889,758,30 clean reads were generated, with high conversion rates (C to G) of 99.54 and 99.47%, respectively. The average read depth of the control and melatonin-treated berries was 12.25 G and 12.42 G, respectively, accompanied by mapping rates of 54.33 and 54.31% (Table S1). A total of 1,169,852 and 1,008,894 methylated cytosines (mCs) were identified in the control and melatonin-treated grapes, respectively. In the control/melatonin treatments, 42.10%/40.52% mCs were identified at CG sites, 30.35%/29.47% at CHG sites and 27.53%/29.99% at CHH sites (H = A, T, or C) (Table S1).

**Fig. 2:**
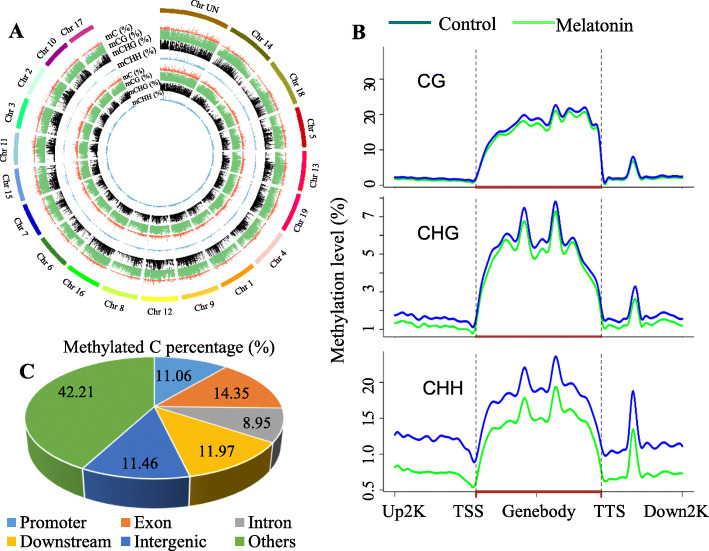
Methylation levels of different chromosomes and genomic regions in ‘Merlot’ berries. **a** The outermost bold lines indicate different chromosomes and their lengths at a 50 kilobase resolution. Red, green, black and blue peak shape diagrams indicate the methylation levels of mC, mCG, mCHG and mCHH, respectively, at different chromosome sites, and the peak height indicates the methylation level. The second to fifth circles are from control berries, and the sixth to ninth circles are from melatonin-treated berries. **b** Changes in the levels of CG, CHG and CHH methylation in melatonin-treated berries compared to the control. TSS, transcription start site. TTS, transcription termination site. Up2K and Down2K represent the 2000 bp upstream of the TTS and downstream of the TTS, respectively. **c** Percentages of differentially methylated cytosines in different genomic regions

**Fig. 3:**
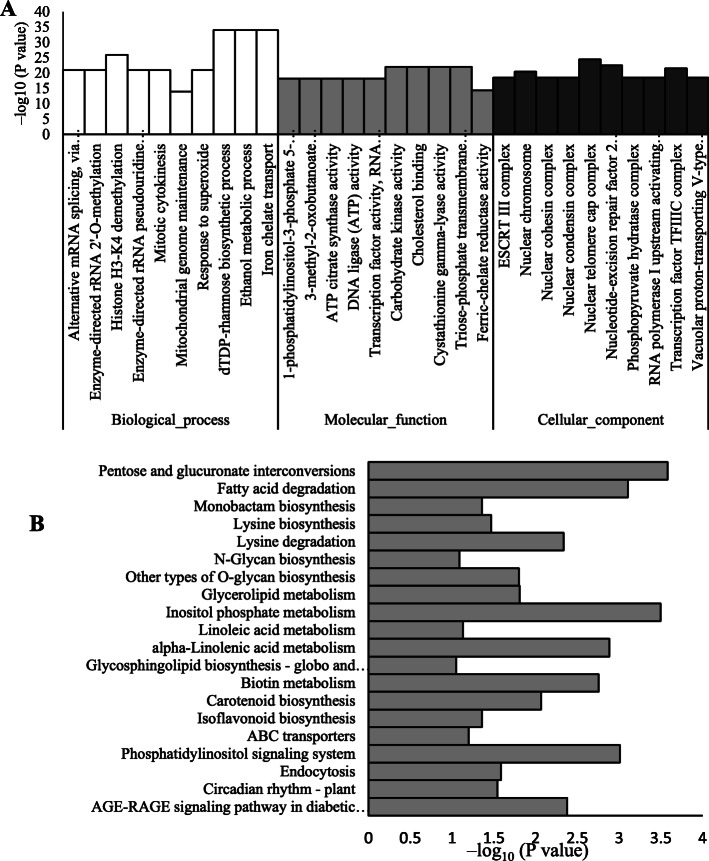
Analysis of the GO (**a**) and KEGG enrichment (**b**) of DMR-associated genes. *P* values are corrected to –log_10_ (*P* values) ranging from 0 to infinity, and a lower *P* value (i.e., a greater –log_10_ (*P* value)) indicates a higher intensity. The top 10 GO enrichments and the top 20 enriched KEGG pathways are displayed in this paper

The control and melatonin-treated berries showed very similar mC profiles on 19 chromosomes (Fig. 2a). The three types of mCs exhibited similar distribution patterns on the same chromosome. In contrast, the different chromosomes showed varying methylation levels and mC distributions. For example, Chrs 16 and 8 possessed the highest and lowest levels of methylation, respectively, among the chromosomes; a high-mC region occurred around the central position of Chrs 4, 6, and 7, while high methylation levels were found on one side of Chrs 18, 1, 8 and 17 (Fig. 2a). Additionally, a high methylation level was detected in the CG context compared to the CHG and CHH contexts in the control and melatonin-treated berries (Fig. 2b). The comparison of methylation levels between the control and melatonin-treated berries revealed that a higher percentage of differentially methylated cytosines in exons than in promoters, introns and downstream regions (Fig. 2c). Additionally, the gene body generally showed a much higher methylation level than its upstream and downstream regions (Fig. 2b). Moreover, melatonin greatly reduced methylation levels in CHH contexts in the upstream, gene body and downstream regions of genes. In contrast, melatonin widely decreased methylation levels in CHG contexts to a lesser extent in various gene regions and led to a slight decline in CG methylation levels only in gene bodies (Fig. 3b).

### Gene ontology (GO) and KEGG enrichment of differentially methylated region (DMR)-related genes

A total of 25,125 DMRs were detected (fold change ≥4 or ≤ 0.25, *P* < 0.05) between the control and melatonin-treated berries. Compared to the control, the methylation levels of 9000 and 16,125 DMRs were increased and decreased, respectively, by melatonin in grape berries. The total DMRs included 6517 DMR-associated genes in upstream, exon, intron and downstream regions (Table S2).

GO enrichment of the DMR-associated genes showed that the three most significantly altered biological processes were dTDP-rhamnose biosynthetic processes, ethanol metabolic processes and iron chelate transport. Enzyme-directed rRNA 2′-O-methylation and alternative mRNA splicing via the spliceosome were also significantly changed (Fig. 3a). The molecular functions of the DMR-associated genes primarily included carbohydrate kinase activity, cholesterol binding, triose-phosphate transmembrane transporter activity and cystathionine gamma-lyase activity. Most of the DMR-associated proteins were located in the nucleus (Fig. 3a). KEGG pathway analysis indicated that the three most significantly changed pathways were pentose and glucoronate interconversion, inositol phosphate metabolism, and fatty acid degradation. Additionally, the metabolism of amino acids, including lysine, linoleic acid and alpha-linolenic acid, and phenolics, including carotenoids and isoflavonoids, was significantly altered (Fig. 3b).

### Melatonin treatment alters global gene expression patterns in ‘merlot’ grape berries

**Fig. 4:**
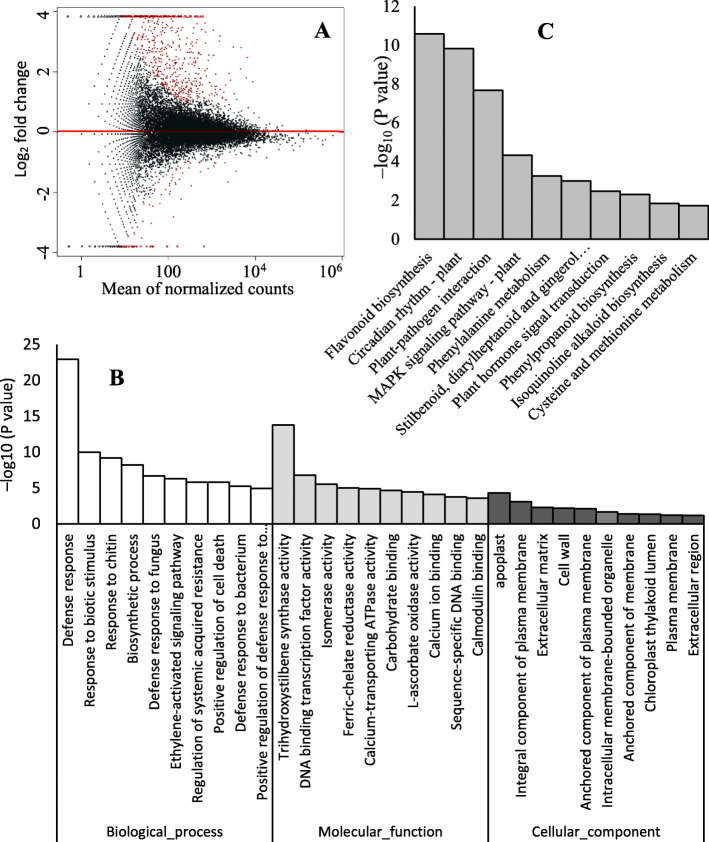
Analysis of GO and KEGG enrichment of differentially expressed genes (DEGs) between the control and melatonin-treated ‘Merlot’ berries. **a** Differential transcript abundance between control and melatonin-treated berries. Red dots indicate differentially expressed genes. **b** GO clustering analysis of DEGs based on their functional enrichment. The top 10 GO enrichments are displayed. **c** KEGG enrichment analysis of DEGs. The top 10 enriched pathways are displayed. *P* values are corrected to –log_10_ (*P* values) ranging from 0 to infinity, and a lower *P* value (i.e., greater –log_10_ (*P* value)) indicates a higher intensity

RNA-Seq was employed to detect the transcript abundance of the control and melatonin-treated berries. A total of 3551 differentially expressed genes (DEGs) (|log_2_FC| > 1, *P* < 0.05) were identified in melatonin-treated berries compared to the control. Compared to the control, 2479 genes were upregulated, and 1072 genes were repressed (Table S3, Fig. 4a). GO enrichment demonstrated that the DEGs in the biological process category were primarily related to defense responses, including the responses to biotic stimuli, chitin and fungi. Among molecular functions, the DEGs were mainly involved in trihydroxystilbene synthase activity, DNA binding transcription factor activity and isomerase activity. The inferred proteins of the DEGs were primarily located in the apoplast and plasma membrane (Fig. 4b). KEGG pathway analysis showed that the most significantly altered pathways were flavonoid biosynthesis, circadian rhythm-plant and plant-pathogen interaction pathways (Fig. 4c).

### Interconnection of the DNA methylome and RNA transcriptome

**Fig. 5:**
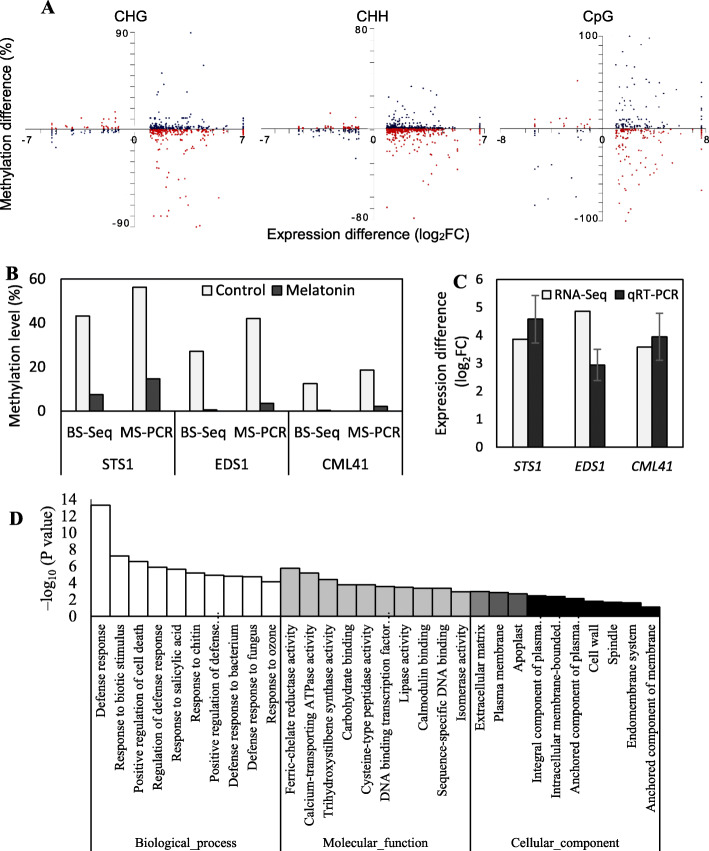
Cross-sectional analysis of differentially methylated genes and DEGs and GO enrichment of the interconnected genes. **a** Cross-analysis was performed between the DMR-related genes and DEGs. Red dots represent negatively related genes, and blue dots represent positively related genes. The y-axis represents the differences in methylation levels (%). The x-axis represents the differences in gene expression. FC, fold change. **b**, **c** Validation of the results obtained from BS-Seq and RNA-Seq via methylation-specific PCR and qRT-PCR analyses of three selected loci. *STS1*, *VIT_216s0100g01100*; *EDS1*, *VIT_217s0000g07370*; *CML41*, *VIT_218s0001g11830*. Functional annotations of the above genes were listed in Table 1. FC (fold change) was calculated using the ratio of the expression in the treated berries to that in the control. **d** GO enrichment of the interconnected genes obtained through the comparison of DMP-related genes and DEGs. The top 10 enriched GO categories are displayed. *P* values are corrected to –log_10_ (*P* values) ranging from 0 to infinity, and a lower *P* value (i.e., greater –log_10_ (*P* value)) indicates a higher intensity

A total of 1626 genes were identified via a cross-analysis of DMR-related genes and DEGs. Among these genes, the expression levels of 886 genes (54.5%) were negatively related to their methylation levels, and 740 genes (45.5%) showed a positive relationship between expression and methylation levels (Table S4, Fig. 5a). Additionally, most of the interconnected genes showed upregulated expression levels in melatonin-treated berries compared to the control (Fig. 5a).

It is well established that methylation at a gene promoter region can inhibit DNA transcription [24]. In this study, we identified 144 differentially methylated promoter (DMP)-related genes exhibiting a negative association between promoter methylation levels and expression levels, among which 133 genes presented upregulated expression and decreased promoter methylation, and 13 genes showed the opposite relationship (Table S5). To verify the association between the levels of promoter methylation and gene expression, three genes involved in flavonoid biosynthesis and plant-pathogen interaction (Table 1) were selected for analysis by methylation-specific PCR (MS-PCR) and qRT-PCR. Compared to the control, the melatonin-treated berries exhibited a low level of promoter methylation and a high level of gene expression (Fig. 5b, c). Almost all of the interconnected genes were related to defense responses, including responses to biotic stimuli, salicylic acid, chitin, bacteria and fungi. Their molecular functions included ferric-chelate reductase, calcium-transporting ATPase, and trihydroxystilbene synthase enzyme activities. The inferred proteins were located in the plasma membrane and outside the plasma membrane (Fig. 5d). Additionally, two KEGG pathways were significantly altered based on these 144 genes: plant-pathogen interaction (*P* = 7.57 × 10^− 6^) and flavonoid biosynthesis (*P* = 7.47 × 10^− 4^).

**Table 1:** The most significantly altered genes in promoter methylation levels (|log_2_FC| > 2 or detected only in control berries) and expression levels (|log_2_FC| > 3 or detected only in melatonin-treated berries)

Gene accession no	Position of DNA methylation	(log_2_FC)		Gene description	Gene function
		Methylation	Expression		
VIT_216s0039g01320	chr16: 724,559–726,558	− 2.06	DT	Phenylalanine ammonia lyase 1 (PAL1)	Flavonoid biosynthesis
VIT_216s0100g01100	chr16: 16,557,734–16,559,733	−2.56	3.86	Stilbene synthase 1 (STS1)	
VIT_200s0203g00220	chrUn: 12,072,634–12,074,633	DC	DT	G-type lectin s-receptor-like serine threonine-protein kinase	Immune response^30^
VIT_212s0059g00160	chr12: 5,071,913–5,073,912	−6.64	DT	Ankyrin repeat-containing protein (ACD6)	Disease resistance and antioxidation metabolism^33^
VIT_217s0000g07370	chr17: 8,237,664–8,239,663	−5.64	4.86	Enhanced disease susceptibility 1 (EDS1)	Plant-pathogen interaction^42,43^
VIT_218s0001g11830	chr18: 10,101,409–10,103,408	−5.64	3.58	probable calcium-binding protein CML41	
VIT_204s0008g07140	chr4: 7,279,720–7,281,719	DC	3.64	Aspartic proteinase CDR1-like	Disease Resistance^36^
VIT_216s0050g01150	chr16: 18052767–18,054,766	DC	4.11	Heat shock protein 83-like	
VIT_204s0044g01420	chr4: 22,951,153–22,953,152	−3.83	4.70	Probable polygalacturonase-like	
VIT_210s0003g05450	chr10: 10098885–10,100,884	−4.32	3.41	Reticuline oxidase-like protein	Response of plants to pathogenic attack ^38^
VIT_219s0014g00470	chr19: 478152–480,151	−3.06	3.07	Leucine-rich repeat receptor-like serine/threonine-protein kinase	MAMP-triggered innate immunity ^38^
VIT_210s0003g01220	chr10: 2,552,768–2,554,767	−4.32	4.42	Heavy metal-associated isoprenylated plant protein 26	Plant responses to environmental changes^35^
VIT_205s0077g01540	chr5: 1240180–1,242,179	−2.25	3.44	Pathogenesis-related protein 10	Response to biotic and abiotic stresses^37^
VIT_204s0008g03530	chr4: 2888437–2,890,436	DC	3.69	Ankyrin repeat-containing protein	Unknown
VIT_203s0063g00550	chr3: 4,059,484–4,061,483	DC	6.01	Unnamed protein product	Unknown
VIT_218s0001g08500	chr18: 6,946,257–6,948,256	−2.40	3.51	Unnamed protein product	Unknown
VIT_200s0270g00120	chrUn: 19,991,241–19,993,240	−4.32	4.59	Kunitz-type trypsin inhibitor	Unknown

*DC* detected only in control berries, *DT* detected only in melatonin-treated berries, *FC* fold change

In addition, the gene functions of the 17 genes with the largest changes in expression (|log_2_FC| > 3 or detected only in melatonin-treated berries) and methylation levels (|log_2_FC| > 2 or detected only in control berries) in the promoter region are listed. Among these genes, *PAL1* and *STS1* are responsible for flavonoid biosynthesis. The functions of four of the genes are unknown. The remaining genes including *EDS1* and *CML41* are involved in disease resistance and/or abiotic stress responses (Table 1).

### Melatonin and DNA methylation inhibitor treatments increase disease resistance and flavonoid accumulation in grape berries

**Fig. 6:**
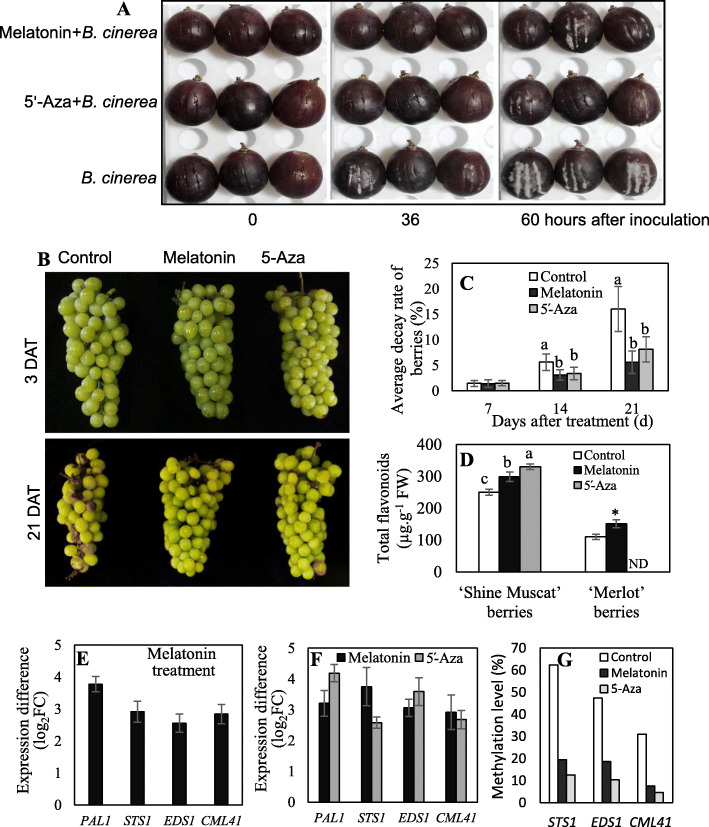
Effects of melatonin and 5́-Aza on disease resistance, flavonoid accumulation, transcripts and DNA methylation of the genes involved in ‘Merlot’ and/or ‘Shine Muscat’ berries. **a** Mycelial growth on *B. cinerea*-inoculated ‘Merlot’ berries treated with or without melatonin and 5́-Aza. **b** Phenotypes of the control and treated ‘Shine Muscat’ berries with melatonin and 5́-Aza. Decayed berries were removed at 21 DAT. **c** The average decay rate of ‘Shine Muscat’ berries determined at 21 DAT. **d** Flavonoid content in ‘Merlot’ and detached ‘Shine Muscat’ berries at 3 DAT. ND, not detected. **e**, **f** Expression difference in ‘Merlot’ (**e**) and detached ‘Shine Muscat’ berries (**f**) at 3 DAT. FC (fold change) was calculated using the ratio of the expression in the treated berries to that in the control. *PAL1*, *VIT_216s0039g01320*; *STS1*, *VIT_216s0100g01100*; *EDS1*, *VIT_217s0000g07370*; *CML41*, *VIT_218s0001g11830*. Functional annotations of the above genes were listed in Table 1. **g** DNA methylation in ‘Shine Muscat’ berries at 3 DAT

To investigate whether melatonin affected the disease resistance of grape berries, 50 μM melatonin treatment of detached ‘Merlot’ and ‘Shine Muscat’ berries was performed. Melatonin clearly decreased mycelial growth on the surface of wounded ‘Merlot’ berries inoculated with *Botrytis cinerea* (*B. cinerea*) compared with the control berries (Fig. 6a). Melatonin decreased the decay rate of ‘Shine Muscat’ berries without inoculation with *B. cinerea* at 14 and 21 DAT (Fig. 6b, c). Additionally, the commonly used DNA methylation inhibitor 5́-azacytidine (5́-Aza) was applied in this study [19]. The application of 5́-Aza led to similar effects on mycelial growth of *B. cinerea* and berry decay rate compared with melatonin (Fig. 6a-c). Therefore, melatonin and 5́-Aza increased disease resistance of grape berries. On the other hand, the effects of melatonin and 5́-Aza on the flavonoid content were determined. Melatonin and 5́-Aza increased the flavonoid content of the ‘Merlot’ and/or detached ‘Shine Muscat’ berries at 3 DAT (Fig. 6d).

Additionally, melatonin upregulated the expression levels of *PAL1*, *STS1*, *EDS1* and *CML41*, which are related to flavonoid biosynthesis or plant-pathogen interaction (Table 1), in the ‘Merlot’ berries at 3 DAT (Fig. 6e). Both melatonin and 5́-Aza increased the expression levels of the above four genes in the detached ‘Shine Muscat’ berries at 3 DAT (Fig. 6f). Moreover, both melatonin and 5́-Aza decreased the methylation levels in the promoter regions of *STS1*, *EDS1* and *CML41* (Fig. 6g). Collectively, melatonin and 5́-Aza increased disease resistance and flavonoid accumulation of grape berries, which might be involved in the upregulation of gene expression caused by the demethylation of the promoter regions.

### The melatonin-induced increase in the promoter transcription-driving capacity is negatively regulated by DNA (cytosine-5)-methyltransferase 1 (MET1)

**Fig. 7:**
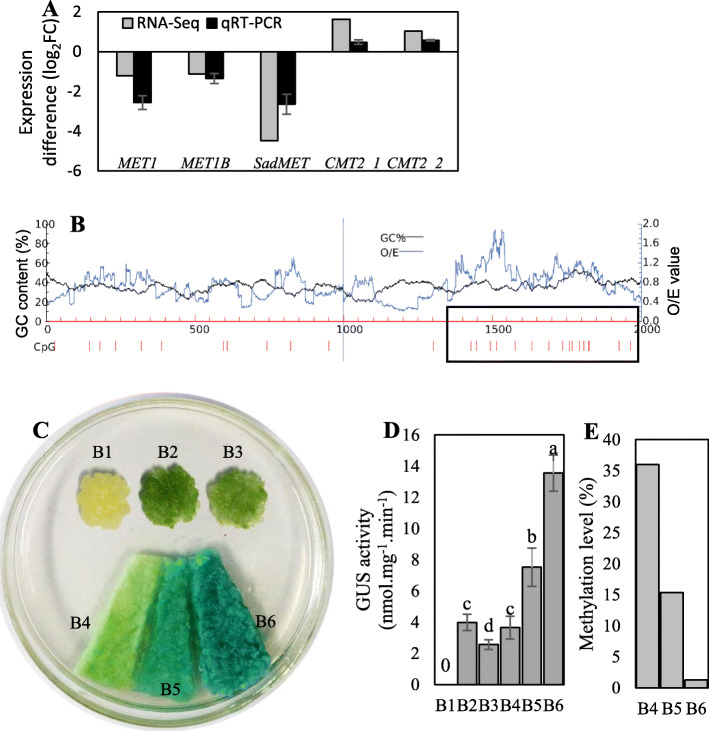
Transcription capacity of the *EDS1* promoter to drive GUS expression in the presence of melatonin and/or MET1. **a** Expression difference of the five selected genes in ‘Merlot’ berries at 48 HAT from RNA-Seq and qRT-PCR analyses. *MET1*, *VIT_212s0035g01770*; *MET1B*, *VIT_212s0035g01755*; *SadMET*, *VIT_214s0006g02170*; *CMT2_1*; *VIT_216s0039g02470*; *CMT2_2*, *VIT_216s0039g02460*. **b** Prediction of methylated cytosine in the *EDS1* promoter (http://www.urogene.org/cgi-bin/methprimer2). O/E values indicate the ratio between the actual value and expected value of the CpG locus. The 800-bp region with a high CpG level indicated by the black box was used as the promoter of *EDS1* (P_eds_) to produce the construct of P_eds_-35S miniGUS. **c** Histochemical analysis of the transcriptional capacity of P_eds_ to drive *GUS* expression in grape calluses agroinfiltrated with different vector constructs: B1, control calluses; B2 and B4, P_eds_-35S miniGUS; B3, P_eds_-35S miniGUS and 35S::MET1; B5, P_eds_-35S miniGUS and 35S::MET1 with melatonin treatment; B6, P_eds_-35S miniGUS with melatonin treatment. **d** Gus activities of grape calluses infiltrated by *Agrobacterium* containing the B1-B6 constructs. **e** DNA methylation level of the *EPS1* promoter, including endogenous DNA and DNA provided by P_eds_- 35S miniGUS, in calluses infiltrated by *Agrobacterium* containing the B4-B6 constructs

As shown in Figs. 5b, c and 6e-g, melatonin treatment decreased the promoter methylation levels and increased the expression levels of the three selected genes. Additionally, the expression levels of *MET1*, *MET1B* and *S-adenosylmethionine-dependent methyltransferase* (*SadMET*) were significantly downregulated by melatonin according to the results of RNA-Seq and qRT-PCRs (Fig. 7a), which was consistent with the melatonin-induced decrease of methylation levels (Fig. 2b). In contrast, the changes in the expression levels of two *CMT2* genes were not associated with the decrease of methylation levels under melatonin treatment (Figs. 2b, 7a). To further elucidate whether melatonin regulated gene expression by modifying promoter methylation, *MET1* (VIT_212s0035g01770) was used to increase the methylation level of the *enhanced disease susceptibility 1* (*EDS1*) (VIT_217s0000g07370) promoter under melatonin treatment. The *Agrobacterium*-mediated transient expression of P_eds_ (800 bp upstream of ATG of *EDS1*, Fig. 7a)-35S miniGUS activated *GUS* expression in grape calluses (Fig. 7c). In contrast to the transformation of P_eds_-35S miniGUS alone, the cotransformation of P_eds_-35S miniGUS and 35S::MET1 decreased the extent of GUS staining and GUS activity (Fig. 7c, d). Therefore, the increased methylation level caused by MET1 reduced the transcription-driving capacity of P_eds_. Additionally, the melatonin-treated calluses expressing P_eds_-35S miniGUS were bluer in color and showed higher GUS activity than control calluses and melatonin-treated calluses cotransformed with P_eds_-35S miniGUS and 35S::MET1 (Fig. 7c, d). Moreover, the above cotransformants showed an increased methylation level of the *EDS1* promoter compared to the calluses expressing P_eds_-35S miniGUS alone under melatonin treatment (Fig. 7e). Collectively, melatonin increased the transcription-driving capacity of P_eds_, at least partially by decreasing the methylation level of P_eds_.

## Discussion

Methylation occurs predominantly in the CG context, followed by CHG and CHH contexts, in plants including rice (CG: 54.7%, CHG: 37.3%, CHH: 12%) [25] and *Arabidopsis* (CG: 24%, CHG: 6.7%, CHH: 1.7%) [26]. A similar pattern was observed in grape berries; however, the methylation rates in the CHG and CHH contexts in grape berries (30.35 and 27.53%, respectively) were higher than those in other species such as rice and *Arabidopsis,* as mentioned above. Additionally, CHH methylation is higher in tomato fruits (13.52–14.20%) than in leaves (8.63%) [27], suggesting the important role of CHH methylation in fruits. The gene body displayed a higher methylation level than its two flanking regions in grape berries (Fig. 2c), while contrary results have been found in citrus fruit [28]. Additionally, it is noteworthy that a global increase in DNA methylation occurs during orange fruit ripening, whereas global demethylation accompanies tomato fruit ripening [20, 27]. Therefore, the methylation patterns of CG, CHG and CHH contexts and their biological significance vary between different species and even in different tissues of the same species, and CHH methylation might play an important role in fruits.

Cytosine methylation is regulated by DNA methyltransferases (MET1, CMT and DRM), demethylases (DME, ROS1, DML) and the chromatin-remodeling factor DDM1 [29]. In plants, the methyltransferase MET is mainly responsible for DNA methylation at the CG site [30], however MET1 also influences CHH methylation [31, 32]. In this study, the significant downregulation of two *MET* genes (VIT_212s0035g01770 and VIT_212s0035g01755) might have contributed to the reduced DNA methylation observed in melatonin-treated berries (Table S3). Methylated DNAs are produced using S-adenosylmethionine as the donor of methyl groups under catalysis by SadMET [33]. The considerable downregulation of *SadMET* (Vit_214s0006g02170) might decrease DNA methylation levels as well (Table S3). The chromoethylase CMT mainly maintains DNA methylation at CHH and CHG sites [34]. Melatonin treatment resulted in much greater effects on CHH methylation than on CG and CHG methylation; therefore, *CMT*s were expected to be downregulated by melatonin. However, only two upregulated *CMT2*s with very low FPKM values were detected in melatonin-treated berries (Table S3), indicating that they might not be the key enzymes responsible for the melatonin-induced decrease in CHH methylation. On the other hand, DNA demethylases such as DML2 are indicated to regulate DNA methylation during tomato and citrus ripening [20]. Differentially expressed DNA demethylases were not detected in the melatonin-treated berries. Taken together, the present results suggested that the melatonin-induced declines in DNA methylation may be related to *MET* and *S-adenosylmethionine-dependent methyltransferase* genes and other underlying mechanisms need to be explored.

Our study showed that melatonin treatment broadly decreased genomic DNA methylation levels and modified gene expression. The correlation between DNA methylation and gene expression is very complex and is influenced by various factors, including tissue type and genomic regions [35]. Only a subset of DMRs are associated with the expression of nearby genes [36]. The role of gene body methylation in modifying gene expression is less well characterized [37]. Limited studies have shown that gene body methylation potentially plays roles in producing new functional gene transcripts by inhibiting RNA splicing [38]. The reduction in gene body methylation (Fig. 2b) and increase in RNA splicing (Table S6) suggested that melatonin increased RNA splicing by decreasing the methylation level of the gene body. In contrast, methylation in a gene promoter region prevents RNA polymerases and transcription factors from binding the promoter, thereby inhibiting DNA transcription [24]. In *Arabidopsis*, reduced DNA methylation contributes to the regulation of pathogen-induced gene expression in hypomethylated and hypermethylated mutants [39]. In this study, the functional annotation of 144 genes exhibiting a negative association between the levels of promoter methylation and gene expression (Table S5) suggested that melatonin might regulate gene expression by modifying promoter methylation and, hence, affect disease resistance and flavonoid biosynthesis in grape berries. This inference was also strongly supported by the similar effects of melatonin and 5́-Aza on the disease resistance, total flavonoid content and changes in promoter methylation and gene expression (Fig. 6). Additionally, the promoter of *EDS1* was used to verify that melatonin increased the transcription-driving capacity of the promotor by decreasing its methylation level (Fig. 7). Collectively, the results indicated that melatonin increased disease resistance and flavonoid biosynthesis at least partially by decreasing the methylation of the gene promoters involved.

Among the DMP-related genes and DEGs showing changes caused by melatonin, two *PAL*s and *STS*s were interconnected in promoter methylation and gene expression (Table 1, Table S5). PAL is the first key enzyme in the phenylpropanoid pathway, in which it catalyzes the biosynthesis of cinnamic acid and provides the initial precursor for other phenolic compounds [40]. Melatonin might broadly affect phenolic metabolism by increasing *PAL* expression. STS is responsible for the biosynthesis of resveratrol [41], and the upregulation of *STS*s suggests a possible role of melatonin in promoting resveratrol biosynthesis in grape berries. On the other hand, melatonin might affect disease resistance through multiple pathways based on the identified interconnected genes (Table S5). For example, EDS1 increases the robustness of the innate immune system by promoting SA biosynthesis [42]. CML41 is required for a complete defense response against bacterial pathogens by enabling Ca^2+^ signaling specificity, a critical component of the immune response [43]. Lectin receptor kinase is related to the activation of immune signaling involving the mitogen-activated protein kinase (MAPK) pathway [44].

## Conclusion

The results indicated that melatonin led to a global decrease in the DNA methylation of grape berries, and the decrease occurred primarily at CHH sites, followed by CHG and CG sites. Simultaneously, melatonin broadly modified gene expression in grape berries. Melatonin increased gene expression at least partially by reducing promoter methylation and thereby promoted disease resistance and flavonoid biosynthesis.

## Methods

### Plant materials and experimental treatments

Grape berries were collected from ‘Merlot’ and ‘Shine Muscat’ vines that were grown at an experimental vineyard in Tai-An City, Shandong Province, China. The ‘Merlot’ berries were used to determine the effects of melatonin on the DNA methylome and RNA transcriptome. Each grape cluster on a vine at 80 days after bloom was soaked for 5 s in a 50 μM melatonin solution plus 0.05% (v/v) Triton X-100. Control berries were treated with 0.05% (v/v) Triton X-100. Detached ‘Shine Muscat’ berries were used to evaluate the effects of melatonin and a methylation inhibitor (5′-Aza) on berry disease resistance and flavonoid accumulation. The control and melatonin treatments were the same as those described above. For 5′-Aza treatment, the detached grape clusters were soaked for 5 s in a 20 μM 5-Aza solution plus 0.05% (v/v) Triton X-100. The treated berries were placed in an environment controlled chamber (20 ± 1 °C, 80% RH, dark) for 21 days. ‘Merlot’ grape calluses were used for promoter assays. The calluses were subcultured on MS medium supplemented with 2.2 mg/L thidiazuron, 10 mg/L picloram and 0.59 g/L 2-(N-morpholino) ethanesulfonic acid at 25 °C under dark conditions. The berries and calluses were collected, rinsed, frozen in liquid nitrogen, and stored at − 70 °C for the determination of DNA methylation, gene expression and other parameters.

### Determination of TSS, titratable acid, relative anthocyanin contents, total flavonoids and average decay rate

Fresh berry pulp was homogenized and filtered. The filtrate was used for the determination of TSS and titratable acid. The TSS content was determined with a PAL-1 digital-display sugar meter (Atago, Tokyo, Japan). Titratable acid was measured by the titration of the filtrate with 0.1 M NaOH to pH 8.3. The results are expressed as mg tartaric acid per g FW. Anthocyanins were extracted and quantified, and the relative anthocyanin content was calculated as reported in our previous study [9]. The total flavonoid content was spectrophotometrically measured using rutin as the standard as described by Dewanto et al. [45]. Berry decay rate was calculated by dividing the number of decayed berries by the total number of berries. The decayed berries were visually evaluated according to the following standard: having slight mildew and moderate shrivel and brown spotting. Measurements were performed with three biological replicates. Each replicate consisted of 10 clusters (approximately 600 berries).

### Melatonin content determination

Melatonin was extracted using a C_18_ solid-phase extraction cartridge (ProElut™; DIKMA, China) according to our previous study [9]. Ten microliter samples were separated using a BEH C_18_ column (Waters, 2.1 mm internal diameter × 50 mm length, and 1.7 μm particle size) in an Acquity UHPLC system (Waters, Milford, MA, USA). Samples were analyzed using a QTof-Micro mass spectrometer (Waters, Milford, MA, USA). The parameters and conditions of the UHPLC-MS analysis were set according to our previous study [12].

### Bisulfite sequencing (BS-Seq) library construction, sequencing and analysis of differentially methylated cytosines

Genomic DNA was extracted from the control and melatonin-treated ‘Merlot’ grape berries. The extracted DNAs were fragmented by sonication to 200–300 bp. After end repair, the adenylation of 3́ ends, and adaptor ligation, the purified ligation products of 275–350 bp were treated with sodium bisulfite using the EZ DNA Methylation Gold Kit (Zymo Research, USA). The resultant DNAs were subjected to paired-end sequencing on the Illumina HiSeq Xten platform (Illumina, Inc., San Diego, CA, USA). Methylome sequencing and analysis were conducted by OE Biotech Co., Ltd. (Shanghai, China). Clean reads of more than 75 bp were obtained by filtering the raw data. Cytosines in the forward strands of the clean reads and the genome were changed to thymidines, and guanines on the reverse strands were changed to adenosines in silico using Bismark software. Then, the clean reads were aligned to the grape reference genome (http://genomes.cribi.unipd.it/DATA/GENOME_12X/).

The detection of methylated cytosine sites and the analysis of DMRs were conducted using MethyKit software. Only the cytosine sites covered by at least 5 reads were used. The true methylated cytosine sites were confirmed by the binomial distribution of methylated and unmethylated cytosines and a false discovery rate (FDR) ≤0.05. A sliding-window approach was used to screen DMRs. The methylation levels (%) of different cytosine sites were integrated in each 1000-bp tiling window, and the integrated data for each window were used for DMR analysis. DMRs were analyzed using the logistic regression mode and identified on the basis of changes in methylation levels (fold change ≥4 or ≤ 0.25) and an FDR ≤ 0.05. Additionally, the region 2000 bp upstream of a transcription start site was taken as the promoter region for the analysis of differentially methylated promoters (DMPs). For DMP analysis, the sliding window was set to 2000 bp, and the other analysis methods were the same as those for DMR analysis. Three biological replicates were performed for the control and melatonin treatment.

### RNA library construction, sequencing and DEG analysis

RNA library construction was performed using the TruSeq Stranded mRNA LT Sample Prep Kit (Illumina, San Diego, CA, USA) according to the manufacturer’s instructions. The RNA libraries were sequenced using the Illumina HiSeq Xten platform (Illumina, Inc., San Diego, CA, USA), and 150-bp paired-end reads were generated. The clean reads were mapped to the grape genome (http://genomes.cribi.unipd.it/DATA/GENOME_12X/) using HISAT2. Unigene expression levels were quantified according to FPKM values, which were calculated using Cufflinks. DEGs between two samples were screened using an absolute log_2_(fold change) ≥1 and an FDR ≤0.05 as the thresholds. Three biological replicates were generated for the control and melatonin-treated berries.

### Bisulfite-RT-PCR (BS-PCR) and quantitative RT-PCR (qRT-PCR)

BS-PCR analysis of three genes was performed to validate the quality of BS sequencing. The DNAs extracted from the control and melatonin-treated berries were treated with bisulfate using an EZ DNA Methylation Gold Kit (Zymo Research, USA). The promoter regions of the above three genes were amplified using specific primers designed with Methyl Primer Express v2.0 (http://www.urogene.org/cgi-bin/methprimer2; Table S7). The BS-PCR products were cloned into pMD19-T (TaKaRa, Dalian, China), and 30 positive clones were sequenced. The sequencing results were used to calculate methylation levels with the online software BiQ Analyzer (http://biq-analyzer.bioinf.mpi-inf.mpg.de/). qRT-PCR was performed using SYBR Green Master-Mix (SYBR Premix EX Taq TM, Dalian, China) on a Bio-Rad iQ5 (Hercules, CA, United States) instrument, and the primers are listed in Table S7.

### Transient transformation of the *EDS1* promoter and *MET1* into grape calluses

The promoter sequence of *EDS1*, 800 bp upstream of ATG, was cloned (see the specific primers in Table S7) and fused upstream of the 35S minimal promoter of pRI101-GUS (Takara, Dalian, China) to generate the P_eds_::35S miniGUS plasmid. The ORF of *MET1* was cloned (see the specific primers in Table S7) and fused downstream of the 35S sequence of pRI101 to generate the 35S::MET1 plasmid. The above two plasmids were introduced into *Agrobacterium* strain GV3101. The *Agrobacterium*-mediated transient transformation of grape calluses was performed according to a previous study [5]. Grape calluses were immersed in an *Agrobacterium* suspension and gently shaken for 20 min. After blotting dry on sterile filter paper, the calluses were transferred to solid MS medium containing 100 μM acetosyringone. After 3 days of coculture in darkness at 28 °C, the calluses were collected and subjected to GUS staining and activity assays. GUS histochemical staining and activity assays were conducted according to a previously reported method as described by Jefferson et al. [46]. GUS activity was expressed as nmol of 4-methylumbelliferone per mg protein per minute.

Inoculation of single detached berries with *B. cinerea*.

The detached ‘Merlot’ berries were soaked for 5 s in a 50 μM melatonin or 20 μM 5′-Aza solution plus 0.05% (v/v) Triton X-100. The berries were then gently wounded with a razor blade to break the surface of the berries. Wounded berries were inoculated by spraying with a suspension of *B. cinerea* (1 × 10^4^ conidia/ml). After inoculation, berries were placed in an environment controlled chamber (20 ± 1 °C, 90% RH, dark).

### Statistical analyses

Statistical analysis was performed using SPSS (v19.0) software. One-way analysis of variance followed by Duncan’s multiple range test was employed.

## Supplementary information

**Additional file 1 Table S1** Details of the bisulfite sequencing, mapping and identification of methylated cytosines (mCs).

**Additional file 2 Table S2** Differentially methylated regions (DMR, fold change ≥4 or ≤ 0.25, *P* < 0.05) and DMR-related genes.

**Additional file 3 Table S3** Differentially expressed genes (DEGs) between the control and melatonin-treated berries (|log_2_FC| > 1, P < 0.05).

**Additional file 4 Table S4** Cross-analysis of DMR-related genes and DEGs.

**Additional file 5 Table S5** Cross-analysis of DMP-related genes and DEGs.

**Additional file 6 Table S6** Alternative splicing frequency statistics for the control and melatonin-treated berries.

**Additional file 7 Table S7** Primers used in this study.

## Data Availability

Full BS-Seq and RNA-Seq data were submitted to the sequence read archive (SRA) of NCBI under BioSample accessions PRJNA603630 and PRJNA603632, respectively (https://www.ncbi.nlm.nih.gov/sra).

## Abbreviations

DAB: Days after bloom
DAT: Days after treatment
DEG: Differentially expressed genes
DMP: Differentially methylated promoter
DMR: Differentially methylated region
EDS1: Enhanced disease susceptibility 1
FC: Fold change
5́-Aza: 5́-azacytidine
GO: Gene ontology
mC: methylated cytosine
HAT: Hours after treatment
MET1: DNA (cytosine-5)-methyltransferase 1
SadMET: S-adenosylmethionine-dependent methyltransferase
TSS: Total soluble solids
